# Dr. Francis Peabody

**Published:** 1897-03

**Authors:** 


### DR. FRANCIS PEABODY.

Dr. Francis Peabody died at Fort Myers, Florida, January 30, 1897. He had been in failing health for a year, and it was hoped a visit to a more genial climate would be of benefit, but this did not have the effect desired, and he passed away as above stated.

Dr. Peabody was born in Boston, Mass., January 22, 1833. He came to Louisville when he was yet a young man and began the study of dentistry with his uncle, Dr. W. H. Goddard, who was one of Louisville's distinguished dentists in the early days. After studying with his uncle for some time he went to Cincinnati, and graduated in the profession from the Ohio Dental College.

He married before the war and went to Tennessee with his wife and two children.

While he took no active part on either side during this conflict, he sympathized with the South, and at one time was imprisoned as a Confederate spy under a mistake.

He remained in practice in Louisville until 1871 when he went to Brazil, practising his profession in Rio Janeiro. He returned to Louisville subsequently and remained there in practice until his death.

Dr. Peabody was not only well known in Kentucky, but in other parts of the country.

He was elected to the faculty of the Louisville College of Dentistry in 1888, and has been head of that institution for the past six years. He has also been president of the Kentucky State Dental Association. He was an active member of the National Association of Faculties, American Dental Association, Southern Dental Association, and Mississippi Valley Dental Association. He was elected president of the Falls City Dental Club last year.

The intimate association which the writer has had for some years in the work of the two dental organizations, the American Dental Association and the National Association of Dental Faculties, has led to a high appreciation of his marked ability in many lines of professional work. His energy combined with the quality of thoroughness in everything undertaken made him a most valuable member in both bodies, but especially so in the latter. The members of this body will learn with deep and lasting regret that they can never again have the privilege of his genial companionship or the benefit of his ripe and cultivated judgment.

The resolutions of the Louisville College of Dentistry and Hospital College of Medicine are appended.

Whereas, Professor Peabody was the champion of many advances in dental education within the period of his professional career. He always insisted upon the intimate relation of dentistry to the profession of medicine, and maintained that the ethics of the medical profession necessarily included the profession of dentistry. He was an enthusiastic student, an able teacher, and a practitioner of rare skill and ability.

*Resolved*, That we deeply deplore his death and feel keenly the loss of his wise counsel and the great loss of his services to this college.

*Resolved*, That in our judgment the dental profession has suffered the loss of one of its ablest members.

*Resolved*, That the college buildings be draped in mourning for thirty days, and that the buildings be closed on the day of the funeral.

*Resolved*, That a committee communicate with the family of the deceased and make suitable preparations for receiving the remains on arrival at the depot and aid in the arrangements for the funeral.

*Resolved*, That the joint faculties and students attend the funeral in a body.

*Resolved*, That we tender to the family of our deceased colleague our profound sympathy and condolence.

*Resolved*, That a copy of this action be furnished the family of the deceased, and that it be made a part of our permanent records.

*Resolved*, That a copy be furnished the press for publication.

Edward M. Kettig,
John A. Larrabee,
Henry B. Tileston,
Thomas Hunt Stucky,
Dudley S. Reynolds,
*Committee.*

